# Contamination of single fluid-filled intragastric balloons with orogastric fluid is not associated with hyperinflation: an ex-vivo study and systematic review of literature

**DOI:** 10.1186/s12876-021-01863-w

**Published:** 2021-07-12

**Authors:** Fadi Hawa, Eric J. Vargas, Andres Acosta, Alison McRae, Fateh Bazerbachi, Barham K. Abu Dayyeh

**Affiliations:** 1 Department of Internal Medicine, St. Joseph Mercy Ann Arbor Hospital, 5333 McAuley Drive, Suite 3009, Ypsilanti, MI 48197 USA; 2 Division of Gastroenterology and Hepatology, Department of Medicine, Mayo Clinic, 200 First Street SW, Rochester, MN 55905 USA; 3 Division of Gastroenterology and Hepatology, St. Cloud Hospital, 1406 6th Ave N, St Cloud, MN 56303 USA

**Keywords:** Adverse outcomes, Bariatrics, Bariatric surgery, Endoscopy, Experimental, Ex-vivo, Gastric balloon, Spontaneous hyperinflation, Obesity, Systematic review

## Abstract

**Background:**

Spontaneous hyperinflation is reported to the Food and Drug Administration as a complication of intragastric balloons. It is postulated that orogastric contamination of the intragastric balloon may cause this phenomenon. We sought to investigate the effects of intentional balloon contamination with gastric contents on intragastric balloon perimeter and contents, whether methylene blue plays a role in preventing spontaneous hyperinflation, and review the available literature on spontaneous hyperinflation.

**Methods:**

Four pairs of balloons with different combinations of sterile saline, orogastric contaminants, and methylene blue were incubated in a 37 °C water bath for six months to simulate physiological conditions with serial measurements of balloon perimeter. Our findings were compared against a systematic review across multiple databases to summarize the available literature.

**Results:**

Balloon mean perimeter decreased from 33.5 cm ± 0.53 cm to 28.5 cm ± 0.46 cm (*p* < 0.0001). No significant differences were seen with the methylene blue group. Only 11 cases were found reported in the literature.

**Conclusions:**

Despite contaminating intragastric balloons with gastric aspirates, hyperinflation did not occur, and other factors may be in play to account for this phenomenon, when observed. Rates of hyperinflation remain under-reported in the literature. Further controlled experiments are needed.

**Supplementary Information:**

The online version contains supplementary material available at 10.1186/s12876-021-01863-w.

## Background

Obesity is the second leading cause of preventable death in the United States, behind tobacco use [1]. Traditional obesity management techniques, such as lifestyle interventions (e.g., diet and physical activity), which remain the foundation of any weight loss program, are often ineffective in inducing clinically significant weight loss alone [2]. On the other hand, bariatric surgery is considered most effective but is reserved for severe obesity classes (class II and III obesity), with low penetration [3].

In this context, endoscopic bariatric therapies (EBTs) emerged as an effective and less-invasive alternative to surgery [4]. This field has the potential to bridge the gap in patients who fail lifestyle interventions or who are intolerant to weight loss pharmacotherapy and are not surgical candidates. Furthermore, in addition to being less-invasive, endoscopic therapies for weight loss are potentially reversible, repeatable, and of lower cost than other medical and surgical alternatives [5].

Among EBTs, the intragastric balloon (IGB) is a minimally invasive, temporary weight loss method that has become one of the most common procedures performed for the less severe cases of obesity (class I and class II obesity with body mass index (BMI) of 30–40 kg/m^2^) [6]. Its efficacy and safety have been demonstrated in the literature for inducing weight loss and reducing obesity-related comorbidities in the adult population [7–10]. Single fluid-filled IGBs were shown to be the most effective type of space-occupying devices in promoting weight loss [11]. The most commonly reported adverse events (AEs) associated with this device are mild accommodative gastrointestinal symptoms, while serious AEs occur in < 1% of cases (e.g., perforation, prosthesis migration) [11–13].

In February 2017, the Food and Drug Administration (FDA) issued an update regarding potential risks with fluid-filled IGBs [14]. It advised close patient monitoring for acute onset of nausea, vomiting, and abdominal pain that could be a sign of spontaneous hyperinflation of the IGB. The FDA defined hyperinflation as the spontaneous filling of IGBs with additional air or liquid while inside a patient’s stomach, typically resulting in the need for early device removal. The onset of symptoms can be as early as seven days and up to 23 weeks after balloon placement. The mechanism behind this AE remains unclear, with reports postulating that orogastric (OG) contamination with microorganisms during IGB insertion is the likely culprit [15, 16]. In this ex-vivo study, we investigate the effects of intentional contamination of the single fluid-filled IGBs with OG contents on balloon perimeter and contents and whether methylene blue (MB) plays a role in preventing spontaneous hyperinflation. A systematic review across multiple databases was also performed to summarize the available literature.

## Methods

An ex-vivo study using eight single fluid-filled IGBs (Orbera, Apollo Endosurgery, Austin, TX, USA) was designed to simulate physiological conditions during device placement for a total of six months. IGBs were filled with a combination of sterile saline, OG contaminants, and MB.

The eight IGBs were divided into four pairs (A1, A2, B1, B2, C1, C2, D1, D2). The first pair (A1, A2) was filled with 650 ml sterile saline; the second pair (B1, B2) was filled with 650 ml sterile saline and inoculated with 3 ml of OG contaminants; the third (C1, C2) and fourth pairs (D1, D2) were filled with 650 ml sterile saline, 3 ml of OG contaminants in addition to 0.5 ml and 2 ml of MB, respectively **(**Table 1).

**Table 1:** Intragastric balloons pairs with respective contents

Intragastric balloons pairs	Contents
A1/A2	650 ml sterile saline
B1/B2	650 ml sterile saline + 3 ml orogastric contaminants
C1/C2	650 ml sterile saline + 3 ml orogastric contaminants + 0.5 ml methylene blue
D1/D2	650 ml sterile saline + 3 ml orogastric contaminants + 2 ml methylene blue

**Fig. 1:**
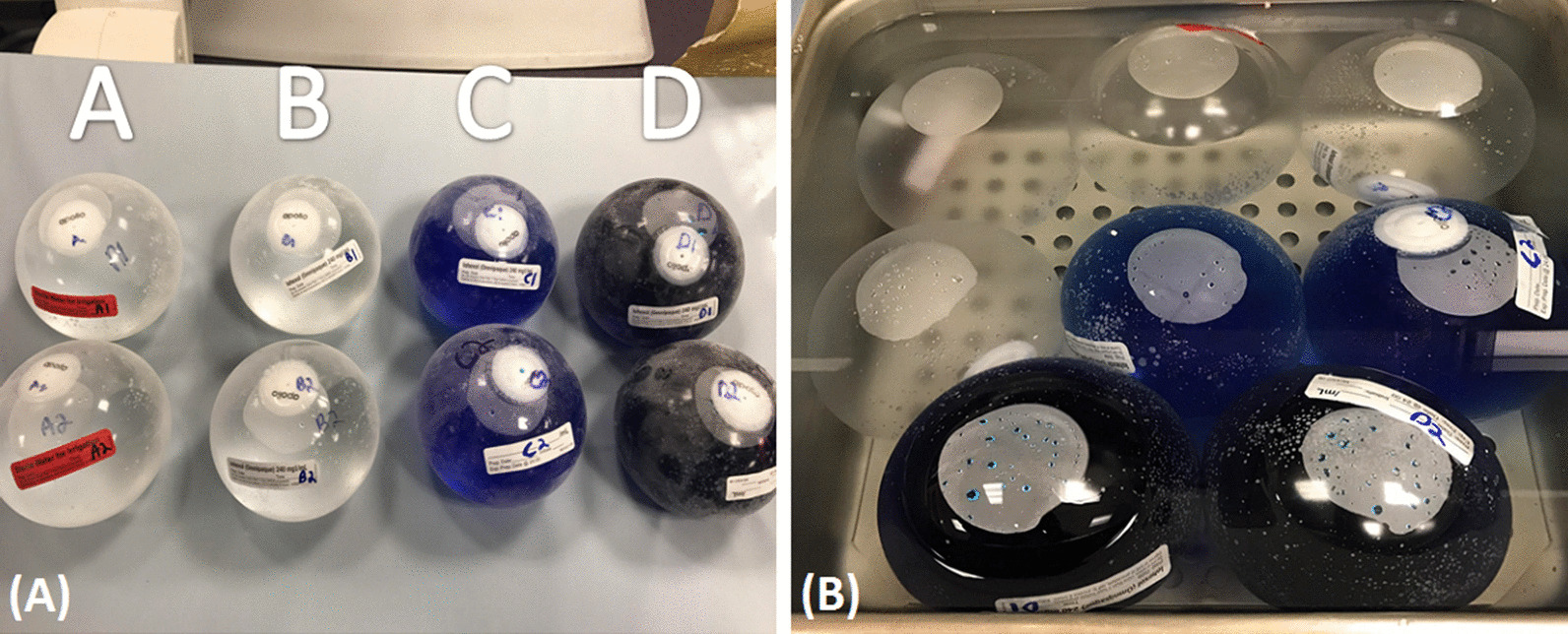
Intragastric balloons. Panel (A): Four pairs of intragastric balloons (A1/A2, B1/B2, C1/C2, D1/D2). Panel (B): Intragastric balloons within the heated water bath with the formation of an initial air bubble

The IGBs were filled using standard equipment delivered with the Orbera balloon system. Each IGB was filled independently without re-use of equipment to avoid cross-contamination. The OG contaminants were obtained during a routine upper endoscopy procedure using standard aspirate techniques. After filling with the respective contents, the IGBs were placed in a water bath incubated in a 37 °C rotating incubator to mimic physiological conditions for the study's planned duration (6 months). Serial balloon perimeter measurements in two dimensions and changes in visual appearance were taken every 7 to 14 days to monitor hyperinflation signs (Fig. 1). The measurements were taken twice by one individual (EJV) using a flexible tape measure.

At the conclusion of the study, the final IGBs mean perimeter was compared to their baseline perimeter using the non-parametric version of the paired *t*-test. In addition, the mean perimeter difference between IGBs with or without MB, and those with and without sterile saline alone were compared. *P* < 0.05 was considered statistically significant.

In order to identify published case reports/series of spontaneous IGB hyperinflation, a medical reference librarian conducted an extensive search of multiple databases without any restriction of language from the inception of the database to February 10, 2021. The data sources and search terms are provided in Additionl file 1. A manual review of the reference lists of relevant publications was done for additional publications. One reviewer (FH) selected the case studies reporting spontaneous IGB hyperinflation and extracted the relevant data onto a standardized form. Data included the year of publication, patient age, sex, and initial BMI, type of IGB used, IGB filling volume, use of MB, hyperinflation symtoms, timing of hyperinflation symptoms post-IGB placement, management approach, management outcome, and IGB fluid culture results.

The quality of the included cases was determined using the methodological quality and synthesis of case series and case reports tool, since all included studies were non-comparative single case reports [17]. According to this instrument, each study is evaluated based on four domains: selection of study groups, ascertainment, causality, and reporting (Additional file 1: Table S1). This resulted in a five-item tool to assess whether the methodological quality of the included studies is good, unclear, or low based on three possible answers for each item (yes, cannot tell, no). This tool has been previously applied with consistency among reviewers [18–22].

## Results

### Results of the ex-vivo study

**Fig. 2:**
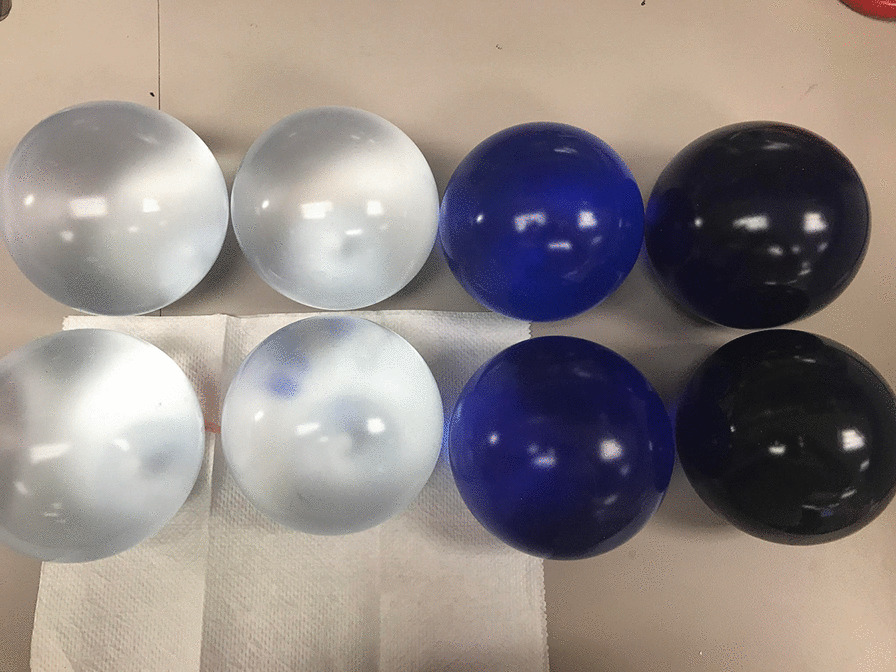
Disappearance of the “air bubble” within 2 weeks into the experiment

Eight (four pairs) IGBs were used in the ex-vivo study, and serial measurements of the perimeter of the balloon were documented over 165 days. Each pair was filled with its unique contents with the formation of an initial “air bubble” that subsequently disappeared two weeks into the incubation period (Fig. 2).

**Fig. 3:**
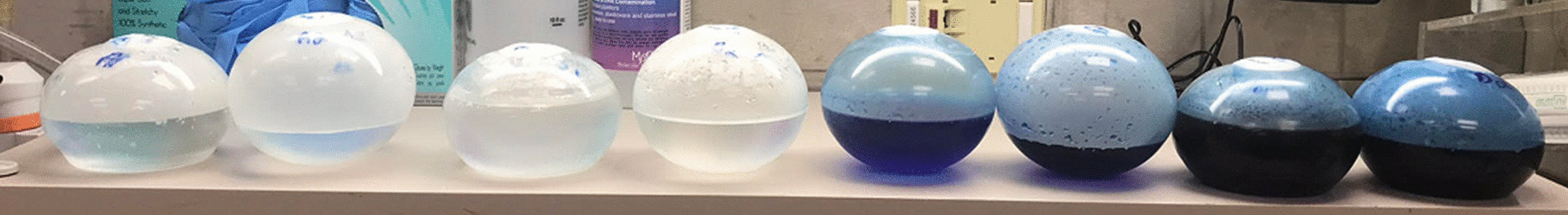
Balloon deflation and development of air-fluid levels at 2 months

All balloons were maintained inside the heated water bath during the study. At two months (56 days), the balloons began to deflate and develop an air-fluid level (Fig. 3).

**Fig. 4:**
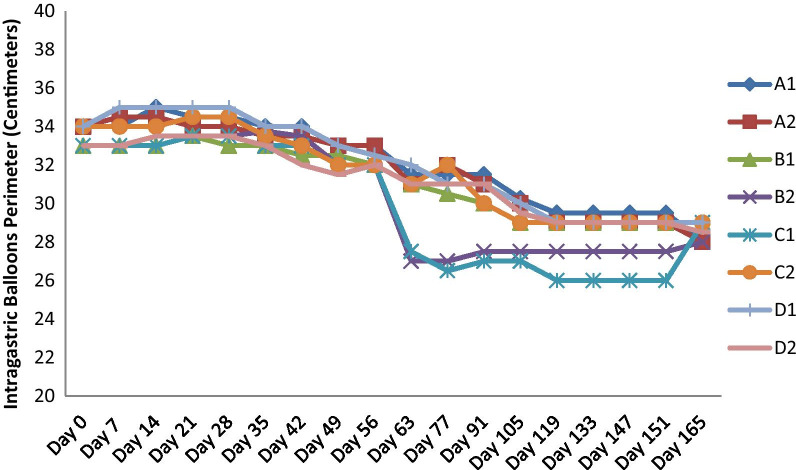
Intragastric balloons perimeter (centimeters) trends over time (days)

Thereafter, balloons continued to decrease in perimeter over the study period (Fig. 4).

At 165 days, the deflation precluded further consistent measurements. Mean perimeter of the balloons dropped from 33.5 cm ± 0.53 cm to 28.5 cm ± 0.46 cm (*p* < 0.0001). The MB groups final mean perimeter was similar to other balloons [28.9 cm ± 0.25 cm vs 28.3 cm ± 0.5 cm (*p* = 0.19)]. The sterile saline group (A1, A2) trended to display a higher final mean perimeter when compared to the other groups with OG contaminants [29.3 cm ± 0.4 cm versus 28.3 cm + 1.3 cm (*p* = 0.07)]. No spontaneous balloon ruptures occurred during the study period.

### Results of the systematic review

**Fig. 5:**
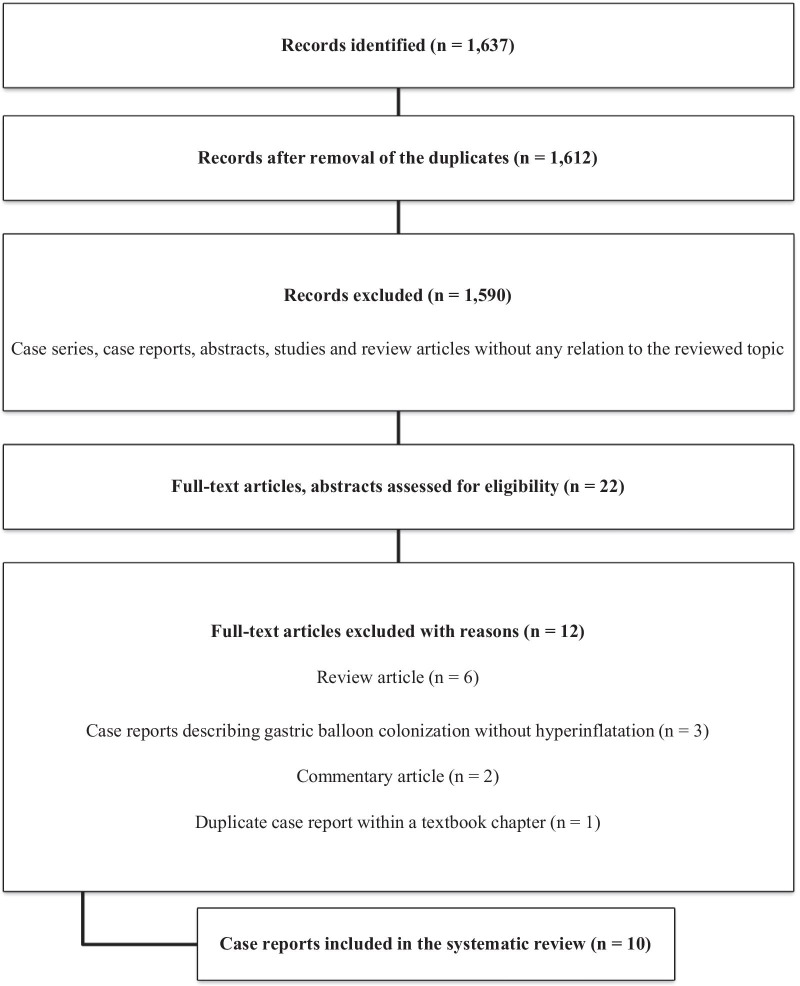
Flow diagram showing the different phases of the systematic review

Figure 5 shows the flow diagram of the systematic review.

A total of 10 publications (11 cases) [15, 23–31] describing spontaneous IGB hyperinflation were identified in the literature despite a reported rate of approximately 2% in post-marketing studies [13]. All identified cases shared a common definition of spontaneous IGB hyperinflation which is consistent with the FDA’s statement [14]. Patient baseline characteristics, type of IGB, clinical presentation and subsequent management are detailed in Table 2.

**Table 2:** Case reports with spontaneous intragastric balloon hyperinflation

First author (year)	Age (years)	Sex	Initial BMI (kg/m^2^)	Type of IGB	Filling volume (ml)	MB use	Hyperinflation symptoms^a^	Timing of symptoms post-placement (months)	Management	Management outcome	IGB fluid culture
Madeira (2013) [28]	45	F	37.4	SB	650	Yes	N/V, AP/AD	4	ER	CR	Klebsiella Pnemoniae, Candida spp.
Patel (2014) [30]	61	F	NR	SB	500	NR	N/V, AP/AD	5	ER	CR	No growth
Marques (2015) [29]	37	F	35	Adj. SB	NR	Yes	N/V, EAP, PP fullness	2	Nystatin and MB refill^b^	CR	Candida spp.
Barola (2017) [26]	45	F	32	SB	650	Yes	N/V, GER	5	ER	NR	Not obtained
De Quadros (2018) [24]	46	F	31.6	SB	NR	NR	EAP/AD, N/V	3	ER	CR	Not obtained
Lopez-Nava (2019) [27]	42	F	31	SB	650	Yes	N/V, AP/AD	2	ER	CR	Candida parapsilosis
Quarta (2019) [25]	62	F	32	SB	NR	NR	EAP	5	ER	NR	Klebsiella spp., Streptococcus, Candida spp.
Barrichello (2020) [23]	53	F	30.2	SB	700	NR	AP	1.5	ER	CR	Not obtained
Basile (2020) [15]	42	F	37	SB	700	NR	AP/AD, N/V, GER	4	ER	NR	Not obtained
Usuy (2020) [31]	62	F	30	Adj.SB	500	Yes	EAP/AD, N/V	3.5	Amoxicillin and MB refill^**b**^	NR	Streptococcus viridans
	38	F	28	Adj. SB	500	Yes	EAP/AD	2.5	ER^**c**^	NR	Candida spp.

Adj. SB: Adjustable single balloon, AP/AD: Abdominal pain/abdominal distention, CR: Complete resolution, EAP: Epigastric abdominal pain, ER: Endoscopic removal, F: Female, GER: Gastroesophageal reflux, IGB: Intragastric balloon, MB: Methylene blue, N/V: Nausea/vomiting, NR: Not reported, PP: Post-prandial, Spp: Species

^a^Balloon hyperinflation was confirmed on upper endoscopy in all cases

^b^These cases utilized adjustable IGBs which contents were emptied and refilled with Nystatin and MB and the other was refilled with Amoxicillin and MB

^c^This case utilized adjustable IGB, which was emptied and refilled with Ceftriaxone and MB without benefit requiring eventual removal after 8 months from placement

The assessment of the case reports’ methodological quality is shown in Additional file 1: Table S2, and the overall evaluation of the methodological quality is shown in Additional file 1: Figure S1. For the selection domain, none of the authors mentioned whether the reported case(s) represented the entire experience of their center. Overall, none of the case reports had a good methodological quality in all domains, with the majority having low or unclear methodological quality.

## Discussion

Intragastric balloon therapy has become one of the most commonly used bariatric endoscopic techniques since its approval by the FDA in 2015, with fluid-filled IGBs being the most effective thus far [6, 11]. IGBs are placed endoscopically for 6 months to promote weight loss, not only by inducing early satiation but also by delaying gastric emptying and restoring satiety between meals [32]. Indeed randomized controlled trials and meta-evidence have demonstrated their higher efficacy in weight loss when combined with dietary interventions and physical activity, compared to the latter alone [11, 33]. The safety of IGBs has also been evaluated in the literature; Genco et al. [34] reported an overall AEs rate of 2.8% in 2515 patients who underwent balloon placement with esophagitis as the most common AE. Furthermore, the American Society for Gastrointestinal Endoscopy bariatric endoscopy task force pooled the rate of AEs after implantation of the Orbera IGB from 68 studies (~ 8500 patients) and found that pain and nausea were the most frequent side effects, occurring in 33.7% of subjects with an early removal rate approximating 7% [7]. This was followed by the Brazilian IGB consensus statement of over 40,000 cases, which reported an AE rate of 2.5%, with the most common being spontaneous hyperinflation of the IGB, with an early removal rate due to intolerance of 2.2% [13].

Worldwide, over 200 AE reports of IGB hyperinflation were received by the FDA with few published reports in the literature [15, 23–31, 35]. Accordingly, the FDA issued an updated letter to providers regarding the potential risk of spontaneous hyperinflation based on the post-approval study of the Orbera IGB [35, 36]. The study found that 6 out of 258 patients (2.3%) experienced balloon hyperinflation; however, the precise mechanism behind this phenomenon remains unknown.

De Souza et al*.* [16] relayed iatrogenic causes as a possible explanation, which can occur at the time of placement if the prosthesis is filled beyond the recommended amount of saline (> 700 ml). Other investigators have conjectured that it may be due to the permeability of the IGB, which results in the entry of fluids and gases by osmosis as the balloon is filled with saline solution [24]. A defective balloon valve allowing air entry or a manufacturing defect of the filling fluid has also been proposed [30, 37]. However, the most widely accepted hypothesis is fungal and/or bacterial contamination of the balloon, a process that can potentially produce gas secondary to fermentation and thus resulting in hyperinflation of the IGB [16].

In our ex-vivo experiment, we aimed to evaluate the effects of purposely inoculating OG contaminants into single fluid-filled IGBs with or without the use of MB, an agent postulated to have antimicrobial effects [38]. The IGBs were placed in a heated water bath to 37 °C to simulate physiological conditions during device placement, and change in the balloons’ perimeter was followed over 165 days (until balloon deflation prevented further measurements). After the study, we noticed that the balloons did not hyperinflate over time but rather decreased in mean perimeter from a mean of 33.5 cm ± 0.53 cm to 28.5 cm ± 0.46 cm (*p* < 0.0001), with the formation of air-fluid levels. Furthermore, the addition of MB did not appear to affect the final IGB mean perimeter (28.9 cm ± 0.25 cm versus 28.3 cm ± 0.5 cm, *p* = 0.19). Similarly, filling the balloon purely with sterile saline only did not result in a different final IGB mean perimeter, compared to the other groups [29.3 cm ± 0.4 cm versus 28.3 cm ± 1.3 cm (*p* = 0.07)].

Intragastric balloons are liable to fungal and bacterial contamination during the passage of the device through the oral cavity secondary to the direct exposure to oral microbiota [39]. Furthermore, the IGB is made of a silicone elastomer, susceptible to colonization by anaerobic bacteria and Candida species. Such colonization results in biofilm formation and subsequent invasion into the balloon contents [40–42]. Saray et al. [43] experimented to compare fungal translocation across silicone tissue expanders with intact injection ports to those with multi-punctured ports. The experiment showed that an intact silicone membrane is impermeable to fungi, while a multi-punctured injection port allows the entry of fungi into the implant. Nonetheless, our systematic review of literature identified multiple case studies reporting asymptomatic microbial colonization of the IGB without balloon hyperinflation [41, 44, 45]. In contrast, others described bacterial or fungal colonization with associated symptomatic spontaneous balloon hyperinflation [25, 27–29, 31]. In combination with the results of our experiment, these observations suggest that IGB contamination and ensuing microbial fermentation may not be the sole cause of hyperinflation, and the presence of additional factors is likely involved.

Silicone is considered a permeable elastomer to gas, water, and protein molecules [42, 46]. In our experiment, we noticed balloon deflation with the simultaneous formation of air-fluid levels. This could be attributed to intra-luminal fluid extrusion or evaporation occurring concurrently with slow air entry into the balloon; hence, it resulted in balloon shrinkage. Alternatively, the relatively higher final mean perimeter of the balloons containing sterile saline only, may suggest a degradative effect of OG contaminants on the balloon structure.

Based on our experiment results, we cannot conclude whether a gas-forming process (fermentation) secondary to OG contamination contributed to the development of the air-fluid levels or not, even though it occurred in all balloons. This observation, and others, point out certain limitations of our study. First, spontaneous hyperinflation is a relatively rare complication of IGB placement. Therefore, using only four pairs of IGBs given the experimental nature of the study may not be adequate to fully investigate the causative factors for spontaneous hyperinflation. Second, the OG contaminants were obtained from random patients without a history of spontaneous IGB hyperinflation. However, the experimentation, being agnostic to the patient from whom the aspirates were collected, simulates the real-life scenario and ignorance of the patient’s fate in term of IGB hyperinflation. Third, the OG contaminants were not cultured prior to inoculating the IGBs or after the conclusion of the study. Furthermore, these contaminants do not fully resemble the gastric contents of patients with IGB placement, largely due to the use proton pump inhibitors (PPIs) post-IGB placement which results in less acidic gastric contents as compared to the OG contaminants used in this experiment. Lastly, it should be noted that the water bath is not a perfect surrogate of post-IGB placement intragastric environment which may have affected the results of the experiment.

We hypothesize that spontaneous IGB hyperinflation occurs in the setting of a multifactorial process rather than secondary to a single culprit. First, the stomach can be considered a confined system, given the intrinsic lower esophageal sphincter tone and the pylorus tone. This anatomy generates an intragastric pressure, and hence favors air diffusion into the balloon rather than fluid extrusion; these circumstances were not available in our water bath environment. Second, the suppressed acid production in the stomach secondary to PPI use in addition to the delayed gastric emptying induced by the IGB can provide a nutritive environment to promote rapid colonization of Candida and bacteria [27, 44, 45]. Third, endoscopic placement of IGB may compromise the silicone elastomer and/or balloon valve integrity, thus resulting in higher permeability of the balloon to air and opportunistic microorganisms [37]. Lastly, bile reflux may still occur into an acid-suppressed stomach while the IGB is in place, which may provide protective effects to fungal growth as has been illustrated by Hsieh and Brock [47].

Irrespective of the etiology of spontaneous hyperinflation, early recognition of this phenomenon in patients with IGB and acute abdomen is paramount. This should be followed by prompt removal of the IGB [24]. De Souza et al. [16] and Usuy et al. [31] proposed adding Nystatin or antibacterial agents to the balloon solution as prophylaxis or treatment for fungal and bacterial colonization. This practice, however, was not recommended by the Brazilian consensus due to a lack of proof that fungal colonization is the culprit behind this phenomenon [13]. Unfortunately, the low incidence of this complication will make it difficult to evaluate in a randomized controlled trial.

## Conclusions

Spontaenous IGB hyperinflation precise pathophysiology remains unclear. However, in combination with the results of our ex-vivo experiment, the current literature suggests that balloon contamination may not be the sole contributor, and the phenomenon is rather a multifactorial process. Additional risk factors and etiologies need to be considered and investigated. Early recognition of this phenomenon and subsequent removal of the IGB is paramount to avoid sinister outcomes.

## Supplementary Information

**Additional file 1**. Supplementary materials, figures, and tables.

## Data Availability

The datasets used and analyzed during the current study (ex-vivo study and systematic review) are available from the corresponding author upon request. Systematic review registration: The systematic review was not registered.

## Abbreviations

AEs: Adverse events
BMI: Body mass index
EBT: Endoscopic bariatric therapies
FDA: Food and drug administration
IGB: Intragastric balloon
MB: Methylene blue
OG: Orogastric
PPI: Proton pump inhibitor

## References

[1] Flegal KM, Carroll MD, Ogden CL, Curtin LR (2010). Prevalence and trends in obesity among US adults, 1999–2008. JAMA.

[2] Acosta A, Streett S, Kroh MD, Cheskin LJ, Saunders KH, Kurian M (2017). White Paper AGA: POWER—practice guide on obesity and weight management, education, and resources. Clin Gastroenterol Hepatol.

[3] Maciejewski ML, Arterburn DE, Van Scoyoc L, Smith VA, Yancy WS, Weidenbacher HJ (2016). Bariatric surgery and long-term durability of weight loss. JAMA Surg.

[4] Kumbhari V, Hill C, Sullivan S (2017). Bariatric endoscopy: state-of-the-art. Curr Opin Gastroenterol.

[5] Kumar N (2015). Endoscopic therapy for weight loss: gastroplasty, duodenal sleeves, intragastric balloons, and aspiration. World J Gastrointest Endosc.

[6] Bazerbachi F, Vargas EJ, Abu Dayyeh BK (2019). Endoscopic bariatric therapy: a guide to the intragastric balloon. Am J Gastroenterol.

[7] ASGE Bariatric Endoscopy Task Force and ASGE Technology Committee, Abu Dayyeh BK, Kumar N, Edmundowicz SA, Jonnalagadda S, Larsen M, et al. ASGE Bariatric Endoscopy Task Force systematic review and meta-analysis assessing the ASGE PIVI thresholds for adopting endoscopic bariatric therapies. *Gastrointest Endosc*. 2015;82:425–438. 10.1016/j.gie.2015.03.1964 26232362

[8] Vargas EJ, Pesta CM, Bali A, Ibegbu E, Bazerbachi F, Moore RL (2018). Single fluid-filled intragastric balloon safe and effective for inducing weight loss in a real-world population. Clin Gastroenterol Hepatol.

[9] Bazerbachi F, Vargas EJ, Rizk M, Maselli DB, Mounajjed T, Venkatesh S (2020). Intragastric balloon placement induces significant metabolic and histologic improvement in patients with nonalcoholic steatohepatitis. Clin Gastroenterol Hepatol.

[10] Bazerbachi F, Vargas EJ, Abu Dayyeh BK (2017). Recent clinical results of endoscopic bariatric therapies as an obesity intervention. Clin Endosc.

[11] Bazerbachi F, Haffar S, Sawas T, Vargas EJ, Kaur RJ, Wang Z (2018). Fluid-filled versus gas-filled intragastric balloons as obesity interventions: a network meta-analysis of randomized trials. Obes Surg.

[12] Abu Dayyeh BK, Edmundowicz SA, Jonnalagadda S, Kumar N, ASGE Bariatric Endoscopy Task Force, ASGE Technology Committee (2015). Endoscopic bariatric therapies. Gastrointest Endosc.

[13] Neto MG, Silva LB, Grecco E, De Quadros LG, Teixeira A, Souza T (2018). Brazilian intragastric balloon consensus statement (BIBC): practical guidelines based on experience of over 40,000 cases. Surg Obes Relat Dis.

[14] U.S. Food & Drug Administration. The FDA alerts health care providers about potential risks with fluid-filled intragastric balloon. 2017. http://www.fda.gov/medical-devices/letters-health-care-providers/fda-alerts-health-care-providers-about-potential-risks-liquid-filled-intragastric-balloons. Accessed 25 May 2020.

[15] Basile P, Marre C, Le Mouel JP (2020). Gastric obstruction secondary to an unexplained hyperinflation of an intragastric balloon. Clin Gastroenterol Hepatol.

[16] de Souza TF, Grecco E, Usuy EN, Galvao Neto M, Silva L, Usuy E, Campos J (2020). Hyperinflated intragastric balloon. Intragastric balloon for weight management.

[17] Murad MH, Sultan S, Haffar S, Bazerbachi F (2018). Methodological quality and synthesis of case series and case reports. BMJ Evid Based Med.

[18] Visrodia KH, Casey B, Clark JW, Hernandez-Barco YG, Hong TS, Jacobson BC (2021). Safety and feasibility of same-session endoscopic ultrasound-guided biopsy and fiducial placement in pancreatic cancer. Tech Innov Gastrointest Endosc.

[19] Siddappa PK, Hawa F, Prokop LJ, Murad MH, Abu Dayyeh BK, Chandrasekhara V (2021). Endoscopic pancreatic duct stenting for pain palliation in selected pancreatic cancer patients: a systematic review and meta-analysis. Gastroenterol Rep.

[20] Bazerbachi F, Dobashi A, Kumar S, Misra S, Buttar NS, Wong Kee Song LM. Efficacy and safety of combined endoscopic cyanoacrylate injection and balloon-occluded retrograde transvenous occlusion (BRTOcc) of gastrorenal shunts in patients with bleeding gastric fundal varices. *Gastroenterol Rep*. 2020. 10.1093/gastro/goaa082 10.1093/gastro/goaa082 PMC8309684 34316370

[21] Bazerbachi F, Leise MD, Watt KD, Murad MH, Prokop LJ, Haffar S (2017). Systematic review of mixed cryoglobulinemia associated with hepatitis E virus infection: association or causation?. Gastroenterol Rep.

[22] Jawoosh M, Haffar S, Deepak P, Meyers A, Lightner AL, Larson DW (2019). Volvulus of the ileal pouch–anal anastomosis: a meta-narrative systematic review of frequency, diagnosis, and treatment outcomes. Gastroenterol Rep.

[23] Barrichello S, de Moura DTH, Hoff AC, Veinert A, Thompson CC (2020). Acute pancreatitis due to intragastric balloon hyperinflation (with video). Gastrointest Endosc.

[24] de Quadros LG, Neto MDPG, Grecco E, de Souza TF, Kaiser Jr RL, Campos JM, et al. Intragastric balloon hyperinsufflation as a cause of acute obstructive abdomen. *ACG Case Rep J*. 2018;5:e69. 10.14309/crj.2018.69 PMC6160611 30280109

[25] Quarta G, Popov VB (2019). Intragastric balloon hyperinflation secondary to polymicrobial overgrowth associated with proton pump inhibitor use. Gastrointest Endosc.

[26] Barola S, Agnihotri A, Chang Chiu A, Kalloo AN, Kumbhari V (2017). Spontaneous hyperinflation of an intragastric balloon 5 months after insertion. Am J Gastroenterol.

[27] Lopez-Nava G, Asokkumar R, Bautista I, Negi A (2020). Spontaneous hyperinflation of intragastric balloon: what caused it?. Endoscopy.

[28] Madeira M, Madeira E, Guedes EP, Mafort TT, Lopes AJ, Moreira RO (2013). Symptomatic bacterial contamination of an intragastric balloon. Gastrointest Endosc.

[29] Marques L, de Souza TF, Grecco E, Neto MDPG, Ramos FM, Verira FM (2015). Proposed treatment of adjustable intragastric balloon contaminated with Candida. Bariatr Surg Pract Patient Care.

[30] Patel KV, Ooi J, Ray S, Griffin N, Oben JA (2014). Abdominal pain following obesity treatment. Gut.

[31] Usuy E, Silva M, Neto MDPG, Grecco E, de Souza TF, de Quadros LG (2020). Antibiotics to prevent relapse of adjustable gastric balloon hyperinflation: feasible for balloon maintenance?. GE Port J Gastroenterol.

[32] Vargas EJ, Bazerbachi F, Calderon G, Prokop LJ, Gomez V, Murad MH (2020). Changes in time of gastric emptying after surgical and endoscopic bariatrics and weight loss: a systematic review and meta-analysis. Clin Gastroenterol Hepatol.

[33] Moura D, Oliveira J, De Moura EGH, Bernardo W, Neto MG, Campos J (2016). Effectiveness of intragastric balloon for obesity: a systematic review and meta-analysis based on randomized control trials. Surg Obes Relat Dis.

[34] Genco A, Bruni T, Doldi SB, Forestieri P, Marino M, Busetto L (2005). BioEnterics intragastric balloon: the Italian experience with 2,515 patients. Obes Surg.

[35] U.S. Food & Drug Administration. UPDATE: Potential risks with liquid-filled intragastric balloons - letter to health care providers. 2020. http://www.fda.gov/medical-devices/letters-health-care-providers/update-potential-risks-liquid-filled-intragastric-balloons-letter-health-care-providers-1. Accessed 25 May 2020.

[36] U.S. Food & Drug Administration. ORBERA PAS (OPAS-001). 2020. http://www.accessdata.fda.gov/scripts/cdrh/cfdocs/cfpma/pma_pas.cfm?t_id=534908&c_id=35. Accessed 25 May 2020.

[37] Ienca R, Al Jarallah M, Caballero A, Giardiello C, Rosa M, Kolmer S (2020). The procedureless Elipse gastric balloon program: multicenter experience in 1770 consecutive patients. Obes Surg.

[38] Ash SR, Steczko J, Brewer LB, Winger RK (2006). Microbial inactivation properties of methylene blue - citrate solution. ASAIO J.

[39] Cho T, Nagao J, Imayoshi R, Tanaka Y (2014). Importance of diversity in the oral microbiota including Candida species revealed by high-throughput technologies. Int J Dent.

[40] da Silveira LC, Charone S, Maia LC, de Araujo Soares RM, Portela MB (2009). Biofilm formation by Candida species on silicone surfaces and latex pacifier nipples: an in vitro study. J Clin Pediatr Dent.

[41] Kotzampassi K, Vasilaki O, Stefanidou C, Grosomanidis V (2013). Candida albicans colonization on an intragastric balloon. Asian J Endosc Surg.

[42] Robinson OG, Benos DJ, Mazzochi C (2005). Spontaneous autoinflation of saline mammary implants: further studies. Aesthet Surg J.

[43] Saray A, Kilic D, Kaygusuz S, Boyunaga H, Ozluk O (2004). Fungal growth inside saline-filled implants and the role of injection ports in fungal translocation: in vitro study. Plast Reconstr Surg.

[44] Coskun H, Bozkurt S (2009). A case of asymptomatic fungal and bacterial colonization of an intragastric balloon. World J Gastroenterol.

[45] Simsek Z, Gurbuz OA, Coban S. Fungal colonization of intragastric balloons. *Endoscopy*. 2014;46 Suppl 1 UCTN:E642-643. 10.1055/s-0034-1390838 25526404

[46] Zhang H, Cloud A. The permeability characteristics of silicone rubber. In: Pilato LA, Kliger HS, Beckwith SW, eds. Sampe fall technical conference global advances in materials and process engineering: 38th international sampe technical conference.DEStech Publications Inc, Lancaster, PA; 2006, pp. 1–10.

[47] Hsieh SH, Brock M (2017). Lipid components of bile increase the protective effect of conjugated bile salts against antifungal drugs. Fungal Biol.

