# Supplementary material for: Contamination of single fluid-filled intragastric balloons with orogastric fluid is not associated with hyperinflation: an ex-vivo study and systematic review of literature
Source: BMC Gastroenterol. 2021 Jul 12;21:286. doi: 10.1186/s12876-021-01863-w (PMC8273974; doi:10.1186/s12876-021-01863-w)
Supplement: Supplementary file 1 — Additional file 1. Supplementary materials, figures, and tables. [file 12876_2021_1863_MOESM1_ESM.docx]

**Supplementary material

Supplementary file 1:** Search strategy for intragastric balloon hyperinflation
 **Supplementary table 1:** Tool for assessment of the methodological quality of case reports and case series
 **Supplementary table 2:** Assessment of methodological quality of included studies
 **Supplementary figure 1:** Evaluation of the methodological quality of the case reports in the systematic review
 **Supplementary table 3:** Checklist of items in reporting a systematic review or meta-analysis (PRISMA checklist)

**Supplementary file 1:** Search strategy for intragastric balloon hyperinflation

**Database(s):** Online databases searched without any restriction of language from the inception of the database to February 10, 2021. PubMed, EMBASE, Cochrane Database, Medline (OVID), Medline (EBSCO), Trip Pro Database, Google Scholar, "gray literature"-government reports, and conference proceedings. Hand searched retrieved articles bibliography lists and relevant journals.

**Search Terms:** "Gastric Balloon/adverse effects"[Majr] ("Equipment Failure"[Mesh]) OR "Equipment Failure Analysis"[Mesh] , ('hyperinflation'/exp OR hyperinflation) AND ('gastric balloon'/exp OR 'gastric balloon') AND balloon AND hyperinflation, 'gastric balloon' AND 'hyperinflation', ('hyperinflation'/exp OR hyperinflation) AND ('gastric balloon'/exp OR 'gastric balloon') AND balloon AND hyperinflation, Intragastric balloon, IGB, gastric balloon, contaminated, contamination, obesity, bariatric surgery, bariatric patients, weight loss, hyperinflation, spontaneous, balloon infection.

**Supplementary table 1:** Tool for assessment of the methodological quality of case reports and case series (adapted from Murad *et al*. [1])

| **Domains** | **Leading explanatory questions** |
| --- | --- |
| **Selection** | Does the patient(s) represent(s) the entire experience of the researchers, or is the selection modality unclear to the extent that other patients with similar presentation may have been missed? |
| **Ascertainment** | 1. Was the exposure sufficiently ascertained?  2. Was the outcome sufficiently ascertained? |
| **Causality** | Were other plausible causes that may account for the observation ruled out beyond a reasonable doubt? |
| **Reporting** | Is the case(s) relayed with adequate details to allow other investigators to replicate the research or to permit practitioners to make inferences related to their practice? |

1. Murad MH, Sultan S, Haffar S et al. Methodological quality and synthesis of case series and case reports. *BMJ Evid Based Med.* 2018; 23: 60-63.

**Supplementary table 2:** Assessment of methodological quality of included case reports/series

**Selection:** (Does the reported case reflect the authors’ entire experience?)

1. Prospective or retrospective study that included all patients within a specified time frame (e.g: 1990-2000), reflecting the authors’ entire experience: Good methodological quality (GMQ)
2. If a study only states several cases without mentioning a time frame as stated in #1: Unclear methodological quality (UMQ)
3. Case reports (1-4 cases) not reflecting the authors’ entire experience: Low methodological quality (LMQ) [1]

**Ascertainment 1:** (Are the procedure components of intragastric balloon (IGB) placement adequately described?)

1. If the study details the indication for IGB placement, type of IGB used, filling volume, and use of methylene blue: GMQ
2. If the study mentions some but not all of above procedure components in #1: UMQ
3. If the study does not mention any of the above procedure components in #1: LMQ

**Ascertainment 2:**(Are the symptoms suggestive of IGB hyperinflation adequately described?)

1. If the study describes a constellation of symptoms including abdominal pain, abdominal distention, nausea, and vomiting, within >3 months from placement of the IGB: GMQ
2. If the study describes a constellation of symptoms including abdominal pain, abdominal distention, nausea, and vomiting, within <3 months from placement of the IGB: UMQ
3. If a study describes only non-specific symptoms such nausea and vomiting, gastroesophageal reflux, or abdominal pain without abdominal distention: LMQ

**Causality:**(Is the IGB placement the sole purpose of the symptoms, beyond a reasonable doubt?)

1. If the study reports complete resolution of symptoms after endoscopic removal of the IGB with a report of the IGB fluid culture results: GMQ
2. If the study reports complete resolution of symptoms after endoscopic removal of the IGB without obtaining an IGB fluid culture: UMQ
3. If the study reports complete resolution of symptoms but management of the hyperinflation did not require IGB removal: UMQ
4. If the study does not report the degree of symptoms improvement after endoscopic removal or the IGB: LMQ

**Reporting:** (Is the endoscopic procedure relayed with adequate details, which allow other practitioners to make inferences related to their practice?)

1. If a study mentions A) type of IGB used, B) IGB filling volume, and C) Use of Methylene Blue: GMQ
2. If a study mentions some but not all of the above procedural components in #1: UMQ
3. If a study does not mention any of the above procedural components in #1: LMQ [1] Case reports were deemed those up to 4 patients based on Abu-Zidan et al.
   Abu-Zidan FM, Abbas AK, Hefny AF. Clinical "case series": a concept analysis. *Afr Health Sci.* 2012; 12: 557-562

| **First Author (Year)** | | **Selection** | **Ascertainment 1** | | **Ascertainment 2** | | **Causality** | **Reporting** |
| --- | --- | --- | --- | --- | --- | --- | --- | --- |
| **Madeira (2013) [1]** | 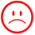 | | 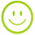 | 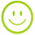 | | 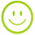 | | 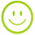 |
| **Patel (2014) [2]** | 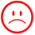 | | 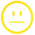 | 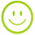 | | 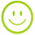 | | 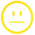 |
| **Marques (2015) [3]** | 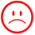 | | 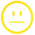 | 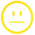 | | 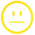 | | 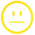 |
| **Barola (2017) [4]** | 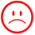 | | **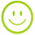** | 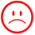 | | 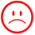 | | 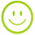 |
| **de Quadros (2018) [5]** | 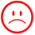 | | **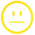** | 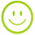 | | 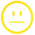 | | 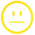 |
| **Lopez-Nava (2019) [6]** | 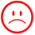 | | **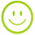** | 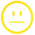 | | 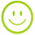 | | 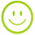 |
| **Quarta (2019) [7]** | 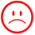 | | **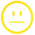** | 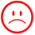 | | 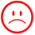 | | 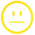 |
| **Barrichello (2020) [8]** | 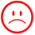 | | 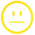 | 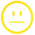 | | 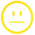 | | 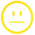 |
| **Basile (2020) [9]** | 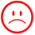 | | 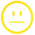 | 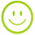 | | 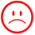 | | 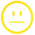 |
| **Usuy (2020) [10]** | 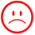 | | 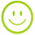 | 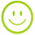 | | 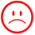 | | 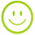 |
| **Total: 10 Studies** | **GMQ 0**  **UMQ 0**  **LMQ 10** | | **GMQ 4**  **UMQ 6**  **LMQ 0** | **GMQ 5**  **UMQ 3**  **LMQ 2** | | **GMQ 3**  **UMQ 3**  **LMQ 4** | | **GMQ 4**  **UMQ 6**  **LMQ 0** |


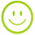
Good methodological quality –
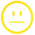
 Unclear methodological quality –
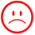
 Low methodological quality

GMQ: good methodological quality – UMQ: unclear methodological quality – LMQ: low methodological quality

**References:**

1. Madeira M, Madeira E, Guedes EP, Mafort TT, Lopes AJ, Moreira RO, et al. Symptomatic bacterial contamination of an intragastric balloon. *Gastrointest Endosc*. 2013;78:360-361.
2. Patel KV, Ooi J, Ray S, Griffin N, Oben JA. Abdominal pain following obesity treatment. *Gut*. 2014;63:364-365.
3. Marques L, de Souza TF, Grecco E, Neto MDPG, Ramos FM, Verira FM, et al. Proposed treatment of adjustable intragastric balloon contaminated with Candida. ***Bariatr Surg Pract Patient Care***. 2015;10:169-172.
4. Barola S, Agnihotri A, Chang Chiu A, Kalloo AN, Kumbhari V. Spontaneous hyperinflation of an intragastric balloon 5 months after insertion. *Am J Gastroenterol*. 2017;112:412.
5. de Quadros LG, Neto MDPG, Grecco E, de Souza TF, Kaiser Jr RL, Campos JM, et al. Intragastric balloon hyperinsufflation as a cause of acute obstructive abdomen. *ACG Case Rep J*. 2018;5:e69.
6. Lopez-Nava G, Asokkumar R, Bautista I, Negi A. Spontaneous hyperinflation of intragastric balloon: What caused it? *Endoscopy*. 2020;52:411-412.
7. Quarta G, Popov VB. Intragastric balloon hyperinflation secondary to polymicrobial overgrowth associated with proton pump inhibitor use. *Gastrointest Endosc*. 2019;90:311-312.
8. Barrichello S, de Moura DTH, Hoff AC, Veinert A, Thompson CC. Acute pancreatitis due to intragastric balloon hyperinflation (with video). *Gastrointest Endosc*. 2020;91:1207-1209.
9. Basile P, Marre C, Le Mouel JP. Gastric obstruction secondary to an unexplained hyperinflation of an intragastric balloon. *Clin Gastroenterol Hepatol*. 2020;18:A16.
10. Usuy E, Silva M, Neto MDPG, Grecco E, de Souza TF, de Quadros LG. Antibiotics to prevent relapse of adjustable gastric balloon hyperinflation: feasible for balloon maintenance? *GE Port J Gastroenterol*. 2020; 28:52-55.

**Supplementary figure 1:** Evaluation of the methodological quality of the case reports in the systematic review

**Supplementary table 3:** Checklist of items in reporting a systematic review or meta-analysis (PRISMA checklist)

| **Section and Topic** | **Item #** | **Checklist item** | **Page/location where item is reported** |
| --- | --- | --- | --- |
| **TITLE** | | |  |
| Title | 1 | Identify the report as a systematic review. | 1 |
| **ABSTRACT** | | |  |
| Abstract | 2 | Provide a structured summary to the systematic review. | 2 |
| **INTRODUCTION** | | |  |
| Rationale | 3 | Describe the rationale for the review in the context of existing knowledge. | 3 |
| Objectives | 4 | Provide an explicit statement of the objective(s) or question(s) the review addresses. | 4 |
| **METHODS** | | |  |
| Eligibility criteria | 5 | Specify the inclusion and exclusion criteria for the review and how studies were grouped for the syntheses. | 6 |
| Information sources | 6 | Specify all databases, registers, websites, organisations, reference lists and other sources searched or consulted to identify studies. Specify the date when each source was last searched or consulted. | 6 and supplementary file 1 |
| Search strategy | 7 | Present the full search strategies for all databases, registers and websites, including any filters and limits used. | Supplementary file 1 |
| Selection process | 8 | Specify the methods used to decide whether a study met the inclusion criteria of the review, including how many reviewers screened each record and each report retrieved, whether they worked independently, and if applicable, details of automation tools used in the process. | 6 |
| Data collection process | 9 | Specify the methods used to collect data from reports, including how many reviewers collected data from each report, whether they worked independently, any processes for obtaining or confirming data from study investigators, and if applicable, details of automation tools used in the process. | 6 |
| Data items | 10 | List and define all outcomes for which data were sought. Specify whether all results that were compatible with each outcome domain in each study were sought (e.g. for all measures, time points, analyses), and if not, the methods used to decide which results to collect. | 6 |
| Study risk of bias assessment | 11 | Specify the methods used to assess risk of bias in the included studies, including details of the tool(s) used, how many reviewers assessed each study and whether they worked independently, and if applicable, details of automation tools used in the process. | 6 and supplementary table 1 |
| Effect measures | 12 | Specify for each outcome the effect measure(s) (e.g. risk ratio, mean difference) used in the synthesis or presentation of results. | Not applicable* |
| Synthesis methods | 13 | Describe the processes used to decide which studies were eligible for each synthesis (e.g. tabulating the study intervention characteristics and comparing against the planned groups for each synthesis. | Not applicable* |
| Reporting bias assessment | 14 | Describe any methods used to assess risk of bias due to missing results in a synthesis (arising from reporting biases). | 6 and supplementary table 1 |
| Certainty assessment | 15 | Describe any methods used to assess certainty (or confidence) in the body of evidence for an outcome. | Not applicable* |
| **RESULTS** | | |  |
| Study selection | 16a | Describe the results of the search and selection process, from the number of records identified in the search to the number of studies included in the review, ideally using a flow diagram. | 8 and figure 5 |
|  | 16b | Cite studies that might appear to meet the inclusion criteria, but which were excluded, and explain why they were excluded. | Figure 5 |
| Study characteristics | 17 | Cite each included study and present its characteristics. | Table 2 |
| Risk of bias in studies | 18 | Present assessments of risk of bias for each included study. | 13, supplementary table 2, and supplementary figure 1 |
| Results of individual studies | 19 | For all outcomes, present, for each study: (a) summary statistics for each group (where appropriate) and (b) an effect estimate and its precision (e.g. confidence/credible interval), ideally using structured tables or plots. | Not applicable* |
| Results of syntheses | 20a | For each synthesis, briefly summarise the characteristics and risk of bias among contributing studies. | 13, table 2, supplementary table 2, and supplementary figure 1 |
|  | 20b | Present results of all statistical syntheses conducted. If meta-analysis was done, present for each the summary estimate and its precision (e.g. confidence/credible interval) and measures of statistical heterogeneity. If comparing groups, describe the direction of the effect. | Not applicable* |
| Reporting biases | 21 | Present assessments of risk of bias due to missing results (arising from reporting biases) for each study assessed. | Supplementary table 2 and supplementary figure 1 |
| Certainty of evidence | 22 | Present assessments of certainty (or confidence) in the body of evidence for each outcome assessed. | Not applicable* |
| **DISCUSSION** | | |  |
| Discussion | 23a | Provide a general interpretation of the results in the context of other evidence. | 13-16 |
|  | 23b | Discuss any limitations of the evidence included in the review. | 15 |
|  | 23c | Discuss implications of the results for practice, policy, and future research. | 16, 17 |
| **OTHER INFORMATION** | | |  |
| Registration and protocol | 24 | Provide registration information for the review, including register name and registration number, or state that the review was not registered. | 17 |
| Support | 25 | Describe sources of financial or non-financial support for the review, and the role of the funders or sponsors in the review. | 18 |
| Competing interests | 26 | Declare any competing interests of review authors. | 17 |
| Availability of data, code and other materials | 27 | Report which of the following are publicly available and where they can be found: template data collection forms; data extracted from included studies; data used for all analyses; analytic code; any other materials used in the review. | 17 |

*Modified From:* Page MJ, McKenzie JE, Bossuyt PM, Boutron I, Hoffmann TC, Mulrow CD, et al. The PRISMA 2020 statement: an updated guideline for reporting systematic reviews. *BMJ.* 2021;372:n71.

*Some of the checklist items were not applicable as the included studies were only case reports and a meta-analysis was not conducted for this systematic review.
